# The effect of a short-term physical activity after meals on gastrointestinal symptoms in individuals with functional abdominal bloating: a randomized clinical trial 

**Published:** 2021

**Authors:** Mohammad Kazem Hosseini-Asl, Erfan Taherifard, Mohammad Reza Mousavi

**Affiliations:** 1 *Department of Internal Medicine, Gastroenterology Ward, School of Medicine, Shiraz University of Medical Sciences, Shiraz, Iran*; 2 *Shiraz University of Medical Sciences, Shiraz, Iran*

**Keywords:** Abdominal bloating, Abdominal distension, Exercise, Physical activity

## Abstract

**Aim:**

The present study aims to compare the effect of a short duration postprandial walking and prokinetic medications on bloating reported by healthy individuals

**Background:**

Abdominal bloating imposes significant clinical, social and economic burden on the healthcare systems; however, treatment of bloating is limited and not effective in all individuals with this symptom. Prokinetic agents are recommended in the treatment of bloating in individuals without underlying disorders traditionally.

**Methods:**

The study participants were randomized into two groups of control and intervention. In the control group, individuals were given daily domperidone plus activated dimethicone as a prokinetic medication, while the subjects in the intervention group were asked to perform a 10-15-minutewalk after each meal. The study duration was 4 weeks, the subjects were re-visited afterwards, and their symptoms was compared before and after the study.

**Results:**

This study consists of 94 individuals including 24 men and 70 women with mean age of 44.47±12.25 years with 49 participants in the control group and 45 participants in the intervention group. Both prokinetic medication use and minimal exercise after meals were associated with significant improvements in the GI tract symptoms such as belching, flatus, postprandial epigastric fullness/ bloating, gas incontinency and abdominal discomfort/pain (p-value <0.001). The changes in the score of the gastrointestinal symptoms from beginning to end of study between the two arms of study were not statistically significant except for postprandial epigastric fullness/ bloating symptoms where the intervention was superior to the use of prokinetics (p-value=0.002).

**Conclusion:**

This study shows that physical activity could be effective in relieving abdominal bloating symptoms. In contrast to other means of treatment proposed for abdominal bloating and its related symptoms, it needs no materials or equipment and can be easily performed by any individual.

## Introduction

 Bloating refers to a subjective feeling of abdominal fullness and pressure which is among the most common gastrointestinal (GI) symptoms. Abdominal distension is another related term characterized by measurable increase in abdominal girth which is commonly associated with bloating (1). Bloating and abdominal distension are complained frequently by patients with functional GI disorders and nonpatient individuals. It is reported that approximately one-third of the general population is suffering from mild to severe degrees of bloating (2). This symptom could significantly affect the individuals’ daily functioning as more than half of those with bloating complain to have a significant reduced quality of life (3). 

Despite clinical and social importance of bloating and economic burden it imposes on the healthcare systems, the pathophysiology of bloating is not completely understood and several gaps remained to be filled(4). Based on the studies conducted to determine the etiopathogenesis, it was revealed that it is likely to be multifactorial including genetic and environmental factors. Although several mechanisms have been considered for bloating such as increased luminal gas production, impaired gas passage and clearance, excessive luminal fluid content, bacterial overgrowth, constipation and altered pelvic floor muscle function, it seems that all share a common component which is dysfunctional gas handling (5). 

Given the poor understanding of the etiology and presence of various underlying mechanisms, each day, different means of treatment are introduced; dietary modifications similar to that used in patients with irritable bowel syndrome (IBS), biofeedback and application of medications, and probiotics are of those interventions reported to be effective (6). However, treatment of bloating is limited and not effective in all individuals with this symptom. Prokinetic agents have been used in the treatment of bloating in individuals without underlying disorders traditionally. It is reported that prokinetic agents reduce excessive amount of luminal gas and relieve bothersome discomfort of bloating (7). Although there is not much evidence on the beneficial effects of prokinetics in the nonpatient individuals with bloating, its application is widely recommended here in Iran, beside dietary and lifestyle modifications. 

Of this lifestyle modification, particularly avoidance of sedentary habits and immobilization are suggested. However, there is limited and contrary evidence regarding the effects of physical activity on bloating. The exact mechanism is not still completely understood; however, several mechanisms has been proposed including additional pressure by physical activity on gut, neurohormonal activation during the activity, etc. Thompson et al. reported that in patients with bloating, the symptoms are gradually increased by activity and afterwards resolves during the resting time (8). Johannesson et al. showed that in patients suffering from IBS, increased physical activity such as walking and cycling is associated with long-term improvement in symptoms such as bloating and postprandial epigastric fullness (9). Consistent with this result, the Digestive System Research Unit of University Hospital Vall d’Hebron showed positive effects of mild physical activity in intestinal gas transit and clearance both in healthy and unhealthy subjects suffering from bloating (10, 11). However, these studies have not yet measured the short-term effects of a simple physical activity such as walking which does not require any equipment on bloating. The present study aims to compare the effect of a short-duration postprandial walking and prokinetic medications on bloating reported by healthy individuals. 

## Methods

**Study participants**

This prospective single-center randomized open trial study was supported by ethics committee of Shiraz University of Medical Sciences. Subjects were enrolled from January 2017 to February 2019. Individuals who complained of bloating were included in the study; they were between 15-75 years old and were diagnosed to have functional abdominal bloating. Rome IV criteria were adopted as diagnostic criteria for functional bloating/distension in the current study which included recurrent episodes of bloating or distension, on average, at least one day per week or abdominal bloating and/or distension predominates over other symptoms for at least three months. Furthermore, based on this criterion, IBS, functional constipation, functional diarrhea, or postprandial distress syndrome should be ruled out. A detailed history taking and physical examination were performed for alarming signs and symptoms. Having history of anorexia, weight loss, alteration in bowel habit, chronic diarrhea and upper/lower GI bleeding over the past 3 months are considered as alarming. Alarming signs in physical examination include jaundice, organomegaly, ascites and temporal wasting. Besides, a series of laboratory data was requested for all the individuals including complete blood counts with differential cell count, erythrocyte sedimentation rate, fasting blood sugar, stool occult blood (for two times), liver and thyroid function tests, blood urea nitrogen, creatinine and electrolytes. Abdominopelvic imaging including ultrasonography and X-ray and upper/lower GI endoscopy was performed in those individuals that was indicated based on their clinical and paraclinical evaluations. Individuals were not included in the study if they had any abnormal laboratory workup indicating an underlying disorder, body mass index ≥40, a history of GI or abdominopelvic surgery or significant coexisting medical condition or medications that affect GI tract motility. Furthermore, pregnant subjects were excluded from the study. 

**Sample size and randomization process**

Based on the previous studies, a Ɵ of 0.5 was considered for the estimation of the minimum sample size of the study(12, 13). Furthermore, minimum sample size required for the study was estimated 40 subjects in each study arms considering type 1 error, one-sided test using the Hochberg procedure at 0.05, statistical power of 80%, and drop-out due to lost to follow-up at 20%,. 

The process of randomization and allocation was performed by an independent external researcher. The participants were randomized into two groups of intervention and control with the allocation rate of 1:1. The randomization process was provided by a web-based randomization application (https://app.studyrandomizer.com/) which provided a block randomization sequence. 

**Study design**

**Figure 1:**
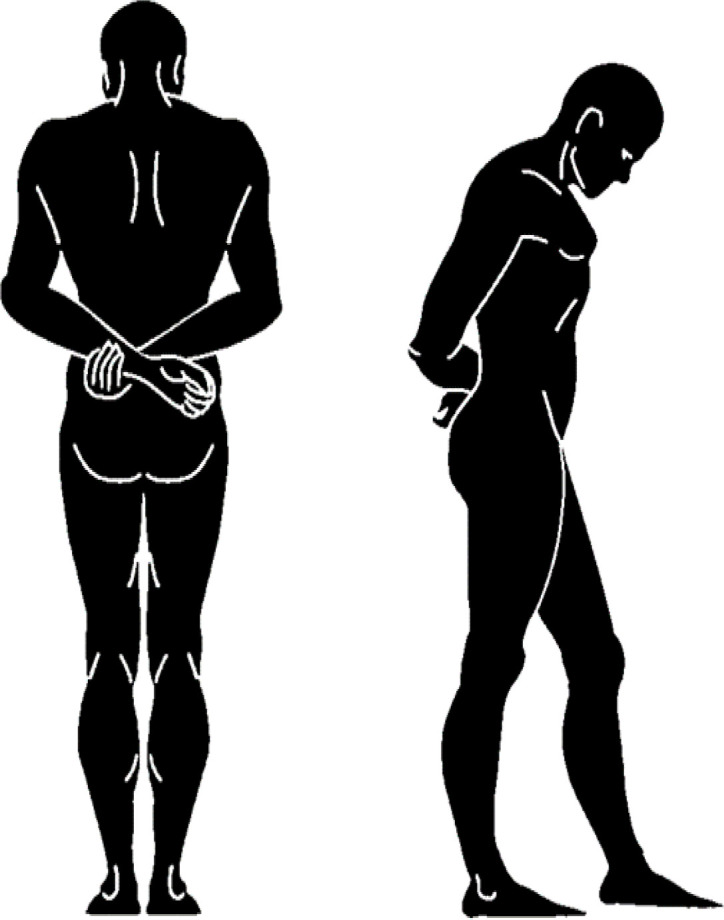
Exercise designed for intervention group after each meal

In the control group, individuals were given daily domperidone (10 mg, 30 mins before lunch and dinner) plus activated dimethicone as a prokinetic medication. However, the subjects in the intervention group were asked to perform a particular exercise after each meal. All the intervention group participants had to install Pedometer Step Counter application on their smartphones (https://play.google.com/store/apps/details?Id=pedometer.steptracker.calorieburner.stepcounter&hl=en). This exercise consists of 10-15 minutes of slow walking (reaching 1000 steps) described in Figure 1, walking with hand clasped together behind the body and flexed neck (around 45 degree) posture without talking meanwhile. The study duration was 4 weeks and afterwards the subjects were re-visited and evaluated for their symptoms. In both groups, it was recommended to take kiwi with skin, sesame oil, prune and damson and avoid gas-producing foods (e.g., onions, broccoli) especially found in salads and piccalilli. Besides, subjects were asked to avoid any change in their medications and to avoid changing their diet during participation in the study.

**Measurements**

The participants were evaluated for subjective feeling of belching, flatus, postprandial epigastric fullness/ bloating, gas incontinency and abdominal discomfort/pain at the baseline using a 5-point Likert scale (1=very mild, 2=mild, 3=moderate, 4=severe, 5=very severe) used in the previous similar works (12, 14, 15). Besides, they were questioned for complaints of clinical symptoms of nausea, vomiting, early satiety and regurgitation. They were thenre-evaluated for these symptoms through the 5-point Likert scale at the end of 4 weeks. 

GI well-being was self-evaluated by subjects at the end of 4 weeks. This scale was adapted from Guyonnet et al. (16) in which the following question was asked from the participants: ‘How do you consider your GI well-being (including all symptoms such as abdominal pain/discomfort, bloating, ﬂatulence/passage of gas, etc.) compared to the period before the beginning of the study?’ The objective was gained using a three-point Likert scale (improved, unchanged or worse) in GI well-being.

**Figure 2:**
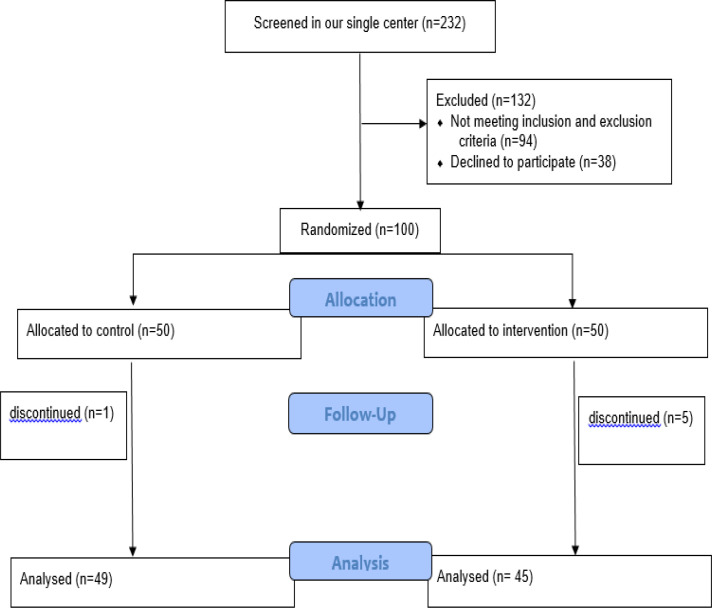
Flowchart of enrollment, allocation, follow-up and analysis of the study subject based on the CONSORT Flow Diagram

Subject’s global change in bloating-related GI symptoms was determined from two means. The first means was the change in the score of 5-point Likert scale between the beginning and the end of study. In the second means, subjects were asked at the end of their participation period in the study to complete the following statement: “Describe how each symptom is now compared with how it was before you began participating in the study using the following Answer Scale: 3=Much better, 2=Better, 1=little better, 0=No change, −1=little worse, −2=Worse, −3=Much worse”. The symptoms assessed in this question included postprandial epigastric fullness/bloating, belching, flatus, abdominal discomfort/pain and gas incontinency. Achieving score below 2 in the answer scale of each of the symptoms was considered as non-significant clinical response and higher score was considered as significant clinical response following the participation in the study (12). 

**Statistical analysis**

Stata statistical software version 16 (statacorp, The United States of America) was used for data analysis. Chi-square statistical method was used to compare clinical symptoms of the participants at baseline and at the end of study between the control and intervention groups. Furthermore, the changes in the score of 5-point Likert scales were compared between the two arms of the study using Chi-square statistical method. The mean change in the scores in each group was also calculated and compared between the groups using independent t-test. P-values of less than 0.05 were considered statistically significant. 

**Table 1.** Baseline characteristics of the subjects. All values are shown in numbers (percentage) expect for age (mean± SD)

Variable	Control group	Intervention group	P-value
Total	49(100)	45(100)	
Age	45.3±12.2	43.5±12.3	0.49
Being female	32(65.3)	38(84.4)	0.03
Ethnicity			
Fars	43(87.7)	39(86.6)	0.87
Non-Fars	6(13.3)	6(12.2)	
Smoker	0(0)	1(2.2)	0.29
Coffee consumer	8(16.3)	6(13.3)	0.64
Alcohol consumer	1(2)	1(2.2)	0.95
Belching			
Very mild/none	14(28.5)	18(40.0)	0.83
Mild	12(24.4)	9(20.0)	
Moderate	12(24.4)	10(22.2)	
Severe	5(10.2)	4(8.8)	
Very severe	6(12.2)	4(8.8)	
Flatus			
Very mild/none	14(28.5)	15(33.3)	0.51
Mild	8(16.3)	9(20.0)	
Moderate	25(51.02)	16(35.5)	
Severe	1(2.04)	2(4.4)	
Very severe	1(2.04)	3(6.6)	
Postprandial fullness/ bloating			
Very mild/none	6(12.2)	2(4.4)	0.72
Mild	14(28.5)	13(28.8)	
Moderate	14(28.5)	15(33.3)	
Severe	7(14.2)	8(17.7)	
Very severe	8(16.3)	7(15.5)	
Gas incontinency			
Very mild/none	11(22.4)	9(20.0)	0.90
Mild	9(18.3)	10(22.2)	
Moderate	14(28.5)	10(22.2)	
Severe	6(12.2)	5(11.1)	
Very severe	9(18.3)	11(24.4)	
Abdominal discomfort/pain			
Very mild/none	13(26.5)	11(24.4)	0.63
Mild	13(26.5)	10(22.2)	
Moderate	12(24.4)	15(33.3)	
Severe	3(6.1)	5(11.1)	
Very severe	8(16.3)	4(8.8)	
Nausea	16(32.7)	18(40)	0.45
Vomiting	3(6.1)	3(6.7)	0.91
Early satiety	13(26.5)	15(33.3)	0.47
Regurgitation	19(38.8)	26(57.8)	0.06

**Ethical issues**

The current randomized trial study was in compliance with the Declaration of Helsinki (1989 revision), and was approved by the ethical committee of Shiraz University of Medical Sciences (License number: IR.SUMS.MED.REC.1399.315). The protocol of the trial study was also registered by Iranian Registry of Clinical Trials (Trial code: IRCT20200310046739N1). The participants of the study were informed of the purpose of the study. They were also assured that the privacy of each participant’s data would be protected. Then, voluntary informed consent was obtained from the participants.

## Results

This RCT study consists of 94 individuals with functional abdominal bloating including 24 men and 70 women with the mean age of 44.47±12.25 years ranging from 16- to 67-year-old. The study included two groups of control and intervention with 49 and 45 participants, respectively (Figure 2). 

There were significant differences in gender between the study groups, control and intervention groups (P-value= 0.03); however, there was no statistically significant differences in other baseline characteristics of the two groups including postprandial epigastric fullness/bloating, belching, flatus, abdominal discomfort/pain and gas incontinency (Table 1). 

In both groups of control and intervention, there were statistically significant differences in the GI tract symptoms such as belching, flatus, postprandial epigastric fullness/ bloating, gas incontinency and abdominal discomfort/pain between the baseline and the end of study, which showed positive effects of both prokinetic medications and physical activity on reducing the gastrointestinal symptoms associated with abdominal bloating(all p-values <0.001). The changes in the score of the gastrointestinal symptoms from beginning to end of study between the two arms of study were not statistically significant except for postprandial epigastric fullness/ bloating symptoms, where the intervention was superior to the control product(p-value=0.002).

Besides, more than half of the participants in each group reported a significant clinical response in individual symptoms of belching, flatus, postprandial epigastric fullness/ bloating, gas incontinency and abdominal discomfort/pain. 

Reported significant improvement of postprandial epigastric fullness symptom from subjects of the intervention group was statistically significantly higher than the improvement experienced by the control group (P-value= 0.028). In other symptoms, there was no significant difference between the control and intervention groups (Table 2). 

**Table 2:** Participants’ self-reported scores at the end of the study

Outcome	Control	Intervention	P.value
Postprandial epigastric fullness/bloating			
Very mild/none	21(42.8)	28(62.2)	0.04
Mild	12(24.4)	12(26.6)	
Moderate	8(16.3)	5(11.1)	
Severe	4(8.1)	0(0)	
Very severe	4(8.1)	0(0)	
Change of postprandial epigastric fullness/bloating score			
0 unit	13(26.5)	7(15.5)	0.01
1 unit	24(48.9)	13(28.8)	
2 units	10(20.4)	16(35.5)	
3 units	2(4.08)	9(20.0)	
Change of postprandial epigastric fullness/bloating*	1.02±0.8	1.6±0.9	0.002
Postprandial epigastric fullness- “significant clinical response”	27(56.2)	35(77.7)	0.02
Belching			
Very mild/none	23(46.9)	30(66.6)	0.07
Mild	18(36.7)	9(20.0)	
Moderate	6(12.2)	1(2.2)	
Severe	1(2.04)	3(6.6)	
Very severe	1(2.04)	2(4.4)	
Change of belching score			
0 unit	24(48.9)	24(53.3)	0.85
1 unit	14(28.5)	14(31.1)	
2 units	9(18.3)	6(13.3)	
3 units	2(4.08)	1(2.22)	
Change of belching*	0.7±0.8	0.6±0.8	0.45
Belching-“significant clinical response”	28(58.3)	26(57.7)	0.95
Flatus			
Very mild/none	26(53.06)	27(60.0)	0.78
Mild	16(32.6)	13(28.8)	
Moderate	7(14.2)	5(11.1)	
Change of belching score			
0 unit	26(53.0)	22(48.8)	0.92
1 unit	14(28.5)	15(33.3)	
2 units	7(14.2)	5(11.1)	
3 units	1(2.04)	1(2.2)	
4 units	1(2.04)	2(4.4)	
Change of flatus*	0.7±0.9	0.8±1.03	0.67
Flatus- “significant clinical response”	34(70.8)	30(66.6)	0.66
Abdominal discomfort/pain			
Very mild/none	25(51.02)	26(57.7)	0.64
Mild	14(28.5)	10(22.2)	
Moderate	5(10.2)	7(15.5)	
Severe	4(8.1)	2(4.4)	
Very severe	1(2.04)	0(0)	
Change of abdominal discomfort/pain			
0 unit	24(48.9)	17(37.7)	0.41
1 unit	18(36.7)	18(40.0)	
2 units	3(6.1)	8(17.7)	
3 units	2(4.08)	1(2.2)	
4 units	2(4.08)	1(2.2)	
Change of abdominal discomfort/pain*	0.7±1.02	0.9±0.9	0.50
Abdominal discomfort/pain- “significant clinical response”	26(54.1)	29(64.44)	0.31
Gas incontinence			
Very mild/none	12(24.4)	11(24.4)	0.85
Mild	10(20.4)	11(24.4)	
Moderate	12(24.4)	11(24.4)	
Severe	8(16.3)	4(8.8)	
Very severe	7(14.2)	8(17.7)	
Change of gas incontinence score			
-2 units	1(2.04)	0(0)	0.64
-1 unit	5(10.2)	2(4.4)	
0 unit	32(65.3)	33(73.3)	
1 unit	10(20.4)	8(17.7)	
2 units	1(2.04)	1(2.2)	
4 units	0(0)	1(2.2)	
Change of gas incontinence*	0.1±0.6	0.2±0.7	0.27
Gas incontinence- “significant clinical response”	11(22.4)	10(22.2)	0.97
Overall perception of GI well-being-“improved response”	35(71.4)	30(66.6)	0.61

*for these variables, mean ± SD is reported in the table.

Associations were assessed between gender and the changes in the score of 5-point Likert scale of postprandial fullness/bloating, belching, flatus, abdominal discomfort/pain and gas incontinency, revealing no statistically significant relationships between them (p-values are 0.25, 0.58, 0.45, 0.44 and 0.91, respectively).

## Discussion

This study shows that both prokinetic medication use and minimal exercise after meals were associated with significant improvements in the GI tract symptoms related to abdominal bloating. Furthermore, except for Postprandial epigastric fullness, subjective symptoms response to exercise among the intervention group subjects was similar to that among the control group subjects. It revealed that minimal exercise after meals is as effective as prokinetic medications and for relieving the postprandial epigastric fullness/ bloating symptoms, the intervention was superior to the control product (p-value=0.002).

Our study was not designed to determine the mechanism by which physical activity results in relieving abdominal bloating and its related symptoms; however, several mechanisms could be attributed to physical activity in order to exhibit this anti-bloating effect. Based on previous studies, it has been shown that exercise could impose a prokinetic effect on gut (18). The abdominal muscle contractions occurring during the physical activity induce somato-autonomic reflex by which the propulsive motor activities of the GI tract are boosted, hence leading to increased luminal contents including fluid and gas transit (18). Besides, individuals in the intervention group would eat small amounts of meal as they know that they should have at least 10 minutes of walking following the meals; however, all participants were recommended to eat less than usual. This lower intake of food results in both the fewer amount of aerophagia and GI production of gas (7); therefore, it is less likely than the control group subjects that these individuals develop gas-related symptoms. Physical activity could also induce increases in intra-abdominal pressure through abdominal compression which is promoted by alteration in the hydrostatic forces’ distribution in the upright position (10, 11). Besides, the exercise that we designed for the intervention group, includes clasping hands together behind back. This body posture could exert an additional pressure onto the thoracic spine and the abdominal cavity. This increased intra-abdominal pressure applies a passive force on luminal gas facilitating its transit and evacuation (19). 

In this study, it was shown that exercise had a significantly better effect than prokinetic medication taking on the postprandial abdominal fullness reported by the participants. Apart from physical activity and its effective mechanisms, another factor could also be responsible for this finding. In individuals without underlying disorder, postprandial fullness is directly related to gastric distension (20). As gastric distension is lower with smaller meals, postprandial abdominal fullness is less perceived in those in the intervention group. 

For intervention, we considered short-term walking with flexed neck and clasped hand together behind back posture. In a study conducted by Dainese et al., intestinal gas transit was evaluated during complete rest and mild physical activity (10). The physical activity designed in this study consisted of 5-minute periods of bicycle pedaling in an arm chair at an angle of 30° to the horizontal. In another study where association between increased physical activity and gas-related symptoms in patients with IBS was assessed, individuals were suggested different exercises at various levels of activity based on their previous physical activity level and experience of exercise (9). However, we asked all the intervention group subjects to follow a particular exercise; besides, in the current study, a kind of physical activity was considered after meals that does not need any special equipment such as that used by Dainese et al. (10) and could be performed easily by anyone. Furthermore, postures requested during the physical activity contribute to positive responses in the abdominal bloating and its related symptoms the intervention group developed following participation. Flexed-neck posture decreases the aerophagia during walking and the clasped hand posture exert a passive pressure on GI tract leading to gas evacuation.

This study has three limitations. First, it was not feasible for our center to monitor the participants in the course of the study and their response to whether prokinetic medication or physical activity was more effective was assessed at the end of study, after 4 weeks of trial. Therefore, it remained unknown during what period of time physical activity left its positive effects. Second, participants’ responses and all other variables considered in this study were all based on the subjective feelings, although majority of studies on the bloating use a similar design. The last limitation stems from an inherent characteristic of abdominal bloating that is multifactorial, and there is no standard regimen for its treatment; therefore, the control treatment considered in this study and other similar RCT studies on bloating is not the standard treatment.

## References

[1] Jiang X, Locke GR, Choung RS, Zinsmeister AR, Schleck CD, Talley NJ (2008). Prevalence and risk factors for abdominal bloating and visible distention: a population-based study. Gut.

[2] Talley NJ, Boyce P, Jones M (1998). Identification of distinct upper and lower gastrointestinal symptom groupings in an urban population. Gut.

[3] Sandler RS, Stewart WF, Liberman JN, Ricci JA, Zorich NL (2000). Abdominal pain, bloating, and diarrhea in the United States: prevalence and impact. Dig Dis Sci.

[4] Iovino P, Bucci C, Tremolaterra F, Santonicola A, Chiarioni G (2014). Bloating and functional gastro-intestinal disorders: where are we and where are we going?. World J Gastroenterol.

[5] Seo AY, Kim N, Oh DH (2013). Abdominal bloating: pathophysiology and treatment. J Neurogastroenterol Motil.

[6] Foley A, Burgell R, Barrett JS, Gibson PR (2014). Management Strategies for Abdominal Bloating and Distension. Gastroenterol Hepatol.

[7] Sullivan SN (2012). Functional abdominal bloating with distention. ISRN Gastroenterol.

[8] Thompson W, Longstreth G, Drossman D, Heaton K, Irvine E, Müller-Lissner SJG (1999). Functional bowel disorders and functional abdominal pain. Gut.

[9] Johannesson E, Ringstrom G, Abrahamsson H, Sadik R (2015). Intervention to increase physical activity in irritable bowel syndrome shows long-term positive effects. World J Gastroenterol.

[10] Dainese R, Serra J, Azpiroz F, Malagelada JR (2004). Effects of physical activity on intestinal gas transit and evacuation in healthy subjects. Am J Med.

[11] Villoria A, Serra J, Azpiroz F, Malagelada JR (2006). Physical activity and intestinal gas clearance in patients with bloating. Am J Gastroenterol.

[12] Ringel-Kulka T, mcrorie J, Ringel Y (2017). Multi-Center, Double-Blind, Randomized, Placebo-Controlled, Parallel-Group Study to Evaluate the Benefit of the Probiotic Bifidobacterium infantis 35624 in Non-Patients With Symptoms of Abdominal Discomfort and Bloating. Am J Gastroenterol.

[13] Whorwell PJ, Altringer L, Morel J, Bond Y, Charbonneau D, O'Mahony L (2006). Efficacy of an encapsulated probiotic Bifidobacterium infantis 35624 in women with irritable bowel syndrome. Am J Gastroenterol.

[14] Saito YA, Schoenfeld P, Locke GR (2002). The epidemiology of irritable bowel syndrome in North America: a systematic review. Am J Gastroenterol.

[15] Marteau P, Le Nevé B, Quinquis L, Pichon C, Whorwell PJ, Guyonnet D (2019). Consumption of a Fermented Milk Product Containing Bifidobacterium lactis CNCM I-2494 in Women Complaining of Minor Digestive Symptoms: Rapid Response Which Is Independent of Dietary Fibre Intake or Physical Activity. Nutrients.

[16] Guyonnet D, Naliboff B, Rondeau P, Mayer E, Chassany O (2013). Gastrointestinal well-being in subjects reporting mild gastrointestinal discomfort: characteristics and properties of a global assessment measure. Br J Nutr.

[17] Hopewell S, Hirst A, Collins GS, Mallett S, Yu L-M, Altman DGJT (2011). Reporting of participant flow diagrams in published reports of randomized trials. Trials.

[18] Keeling WF, Martin BJ (1987). Gastrointestinal transit during mild exercise. J Appl Physiol.

[19] Dainese R, Serra J, Azpiroz F, Malagelada JR (2003). Influence of body posture on intestinal transit of gas. Gut.

[20] Jones KL, Doran SM, Hveem K, Bartholomeusz FD, Morley JE, Sun WM (1997). Relation between postprandial satiation and antral area in normal subjects. Am J Clin Nutr.

